# Supplementary material for: Development of High-Performance Flexible Radiative Cooling Film Using PDMS/TiO2 Microparticles
Source: Micromachines (Basel). 2023 Dec 10;14(12):2223. doi: 10.3390/mi14122223 (PMC10745724; doi:10.3390/mi14122223)
Supplement: Supplementary file 1 [file micromachines-14-02223-s001.zip › micromachines-2754588-supplementary.pdf]

Supplementary Materials: Development of High-Performance Flexible Radiative Cooling Film Using PDMS/TiO_2 Microparticles

Junbo Jung [1], Siwon Yoon [2], Bumjoo Kim [3,4,*] and Joong Bae Kim [3,*]

[1] Department of Mechanical Engineering, Kongju National University, Cheonan 31080, Republic of Korea;
[2] Energy Efficiency Research Division, Korea Institute of Energy Research, Daejeon 34129, Republic of Korea;
[3] Department of Mechanical and Automotive Engineering, Kongju National University, Cheonan 31080, Republic of Korea
[4] Department of Future Convergence Engineering, Kongju National University, Cheonan 31080, Republic of Korea

* Corresponding authors : bumjoo@kongju.ac.kr (B.K.); joongbae.kim@kongju.ac.kr (J.B.K.)

1. Calculated Optical Properties Depending on the Parameters of the FRC Film

The optical properties of the flexible radiative cooling (FRC) film are designed using the Mie-scattering-theory-based Monte Carlo method. This involves calculating the spectral reflectance in the solar spectrum and spectral emittance in the atmospheric window for different parameter cases. Key parameters considered in the design include the microparticle diameter, microparticle volume fraction, and film thickness.

1.1. Diameter of TiO_2 Microparticle

Figure S1a,b present the calculated spectral reflectance in the solar spectrum and the spectral emittance in the atmospheric window depending on the TiO_2 microparticle diameter. For this calculation, the TiO_2 microparticle volume fraction and thickness were fixed at 0.3 and 2 mm, respectively. It can be observed that the spectral reflectance in the solar spectrum differs depending on the diameter of the microparticles. Specifically, as the microparticle diameter decreases, the spectral reflectance decreases in the near-infrared regime. In contrast, the spectral emittance in the atmospheric window exhibits slight differences in the range of 8 μm to 9 μm, but it remains similar in other wavelength ranges regardless of the microparticle diameter.

2.2. Volume Fraction of TiO_2 Microparticle

Figure S2a,b show the calculated spectral reflectance in the solar spectrum and the spectral emittance in the atmospheric window based on the TiO_2 microparticle volume fraction. The calculation parameters were set

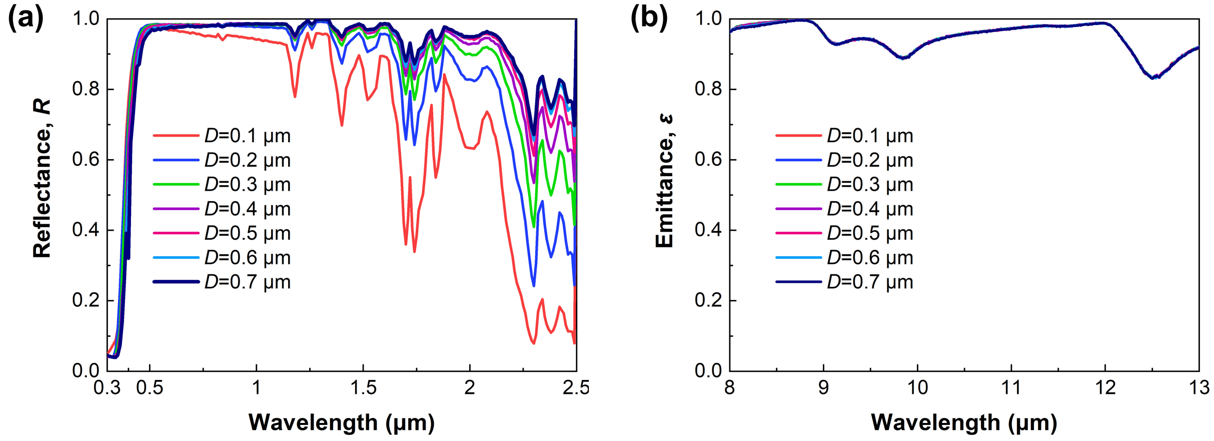

Figure S1. Calculated optical properties depending on the microparticle diameter. (**a**) Spectral reflectance in the solar spectrum. (**b**) Spectral emittance in the atmospheric window.

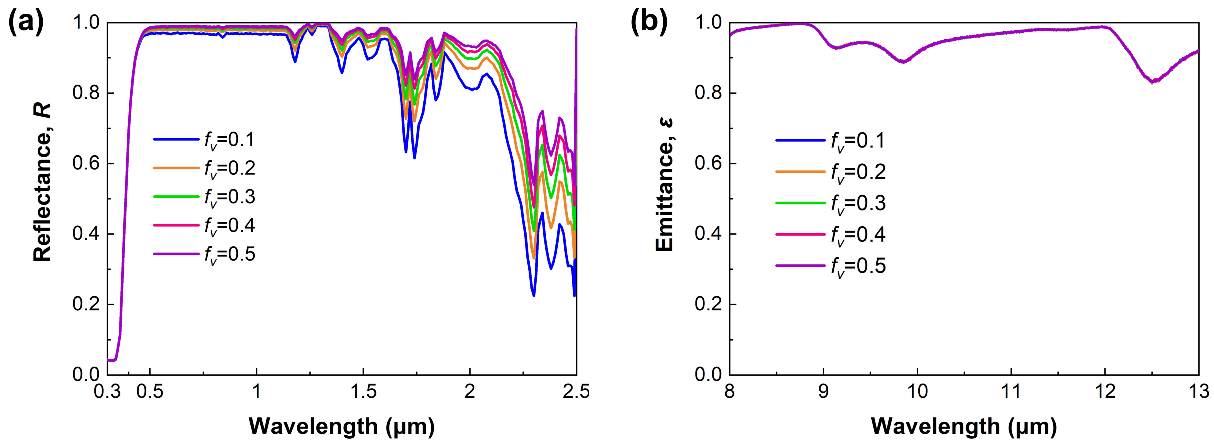

Figure S2. Calculated optical properties based on the microparticle volume fraction. (**a**) Spectral reflectance in the solar spectrum. (**b**) Spectral emittance in the atmospheric window.

to 0.3 µm of the microparticle diameter and 2 mm of the film thickness. The calculated reflectance results in the solar spectrum vary with the microparticle volume fraction, similar to the microparticle diameter in the previous results. In particular, the spectral reflectance in the visible and near-infrared regimes decreases as the volume fraction decreases. On the other hand, the spectral emittance in the atmospheric window shows no discernible differences based on the microparticle volume fraction. Accordingly, the spectral emittance results appear as a single solid line.

3.3. Film Thickness

Figure S3a,b present the calculated spectral reflectance in the solar spectrum and spectral emittance in the atmospheric window. For this case, the TiO_2 microparticle diameter and volume fraction were fixed at 0.3 µm and 0.3, respectively. The spectral reflectance of the 100 µm film thickness is lower than the others in the visible regime, but between 2 and 2.4 µm, the film thickness of 100 µm is the highest. Moreover, the spectral emittance is low at 8–8.4 µm. Therefore, having a thickness of at least 500 µm is necessary to achieve high cooling performance.

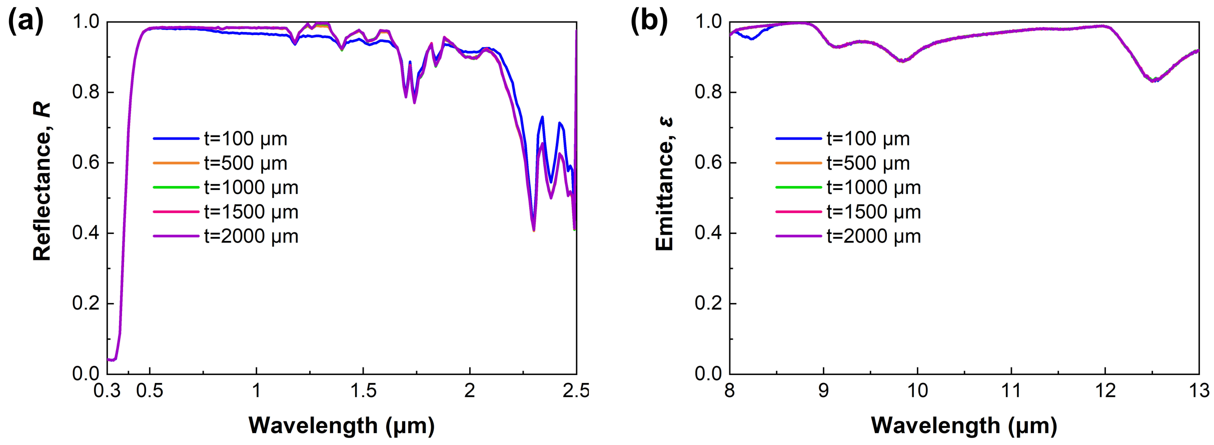

Figure S3. Calculated optical properties depending on the film thickness. (**a**) Spectral reflectance in the solar spectrum. (**b**) Spectral emittance in the atmospheric window.

2. Calculation Method of Cooling Power

The cooling power (P_{net}) is calculated by Equation (S1) and the detailed terms required for this can be expressed as [1, 2]:

$$P_{net} = P_{rad}(T) - P_{atm}(T_{amb}) - P_{sun} - P_{nonrad} \tag{S1}$$

where P_{net} is the net cooling power at the surface temperature of the FRC film T. P_{rad} is the power radiated out by the radiative cooling film and can be determined from Equation (S2):

$$P_{rad}(T) = \int_0^{2\pi} \int_0^{\pi/2} \int_0^{\infty} I_{BB}(T, \lambda)\varepsilon(\lambda, \theta) cos\theta sin\theta d\lambda d\theta d\phi \tag{S2}$$

$I_{BB} = (\frac{2hc^2}{\lambda^5})/[e^{kc/\lambda k_B T} - 1]$ is the spectral radiance of a blackbody at temperature T, where h, c, and k_{BB} are Planck's constant, the speed of light, and the Boltzmann constant, respectively. Here, $\varepsilon(\lambda,\theta)$ is the spectral emittance according to the wavelength and angle of the film, and it is assumed that the spectral absorptance and spectral emittance of the film are the same by Kirchhoff's law. In Equation (S1), P_{atm} means the atmospheric thermal radiation and can be defined as Equation (S3):

$$P_{atm}(T_{amb}) = \int_0^{2\pi} \int_0^{\pi/2} \int_0^{\infty} I_{BB}(T_{amb}, \lambda)\varepsilon(\lambda, \theta)\varepsilon_{atm}(\lambda, \theta) cos\theta sin\theta d\lambda d\theta d\phi \tag{S3}$$

where $\varepsilon_{atm}(\lambda, \theta) = 1 - t(\lambda)^{1/cosa\theta}$ is the angle dependent emittance of the atmosphere.

$$P_{sun} = \int_0^{\infty} I_{AM1.5}(\lambda)\varepsilon(\lambda, \theta_{sun}) d\lambda \tag{S4}$$

is the absorbed power of the incident solar energy by the film where $I_{AM1.5}(\lambda)$ is the spectral solar irradiance, and we use the AM1.5 spectrum [3]. P_{nonrad} is the heat loss due to convection and conduction, and it is simply expressed as the following Equation (S5):

$$P_{nonrad} = h_c(T_{amb} - T) \tag{S5}$$

where h_c is the heat transfer coefficient. The heat transfer coefficient is set to 6 W/m^{2}K within the range corresponding to natural convection [2].

References

1. Hossain, M.M.; Gu, M. Radiative cooling: Principles, progress, and potentials. *Adv. Sci.* **2016**, *3*, 1500360.
2. Chae, D.; Kim, M.; Jung, P.H.; Son, S.; Seo, J.; Liu, Y.; Lee, B.J.; Lee, H. Spectrally selective inorganic-based multilayer emitter for daytime radiative cooling. *ACS Appl. Mater.* **2020**, *12*, 8073–8081.
3. Rephaeli, E.; Raman, A.; Fan, S. Ultrabroadband photonic structures to achieve high-performance daytime radiative cooling. *Nano Lett.* **2013**, *13*, 1457–1461.
